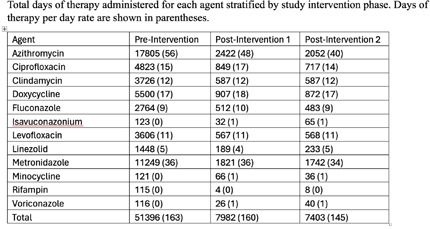# Impact of Optimized IV to PO Antibiotic Conversion on Carbon Emissions

**DOI:** 10.1017/ash.2025.207

**Published:** 2025-09-24

**Authors:** Amulya Mallu

## Abstract

**Background:** Antimicrobials are among the most prescribed medications in the hospital setting and intravenous antimicrobial use is associated with increased carbon emissions due to use of single-use disposable products for packaging, preparation, and administration. IV to PO antibiotic switch has been associated with lower waste and emissions and lower healthcare costs. This project aims to assess the effectiveness of pharmacist interventions in switching from intravenous to oral antibiotics and estimate emissions reductions. **Methods:** Our study population included adult patients hospitalized between October 1, 2023 and September 30, 2024 in one of twelve medical centers operating within a large, integrated healthcare system in Northeast Ohio. The primary intervention phase involved reinforcement of a pre-existing policy within our hospital system allowing pharmacists to convert certain highly bioavailable agents from IV to PO based on clinical criteria. The second intervention phase occurred as part of the hospital response to the nationwide IV fluid shortage beginning at the end of September 2024. We determined the rate of interventions occurring within the pre-intervention period and applied this rate to the intervention cohorts to determine what the expected number of interventions would be during these periods if the interventions would have had no effect. From the total days of therapy for each agent for each period, we estimated the total carbon emissions for each period, using our antimicrobial carbon emissions calculator. **Results:** Compared to the pre-intervention group, the rate ratio for the second intervention phase was 2.97 (95% CI 2.61 to 3.37). The second intervention group rate versus the first intervention rate was 1.49 (95% CI 1.26 to 1.77). Using the difference between the actual and expected number of pharmacy interventions, the potential DOT saved using conservative (assuming 1 pharmacy intervention saves 1 DOT) and liberal (assuming 1 pharmacy intervention saves 3 DOT) estimates was determined, as well as the actual DOT saved based on the antimicrobial administration data. These values were used to calculate the carbon dioxide emissions and equivalents potentially and actually saved (Figure 2). The total actual emissions saved was less than both conservative and liberal estimates for post-intervention phase 1 versus pre-intervention phase, but it surpassed both estimates for post-intervention phase 2 versus pre-intervention phase. **Conclusion:** This is one of the first projects to estimate carbon emission reductions associated with IV to PO antibiotic switching. Future research should focus on identifying further opportunities to promote sustainable policies and measuring their impact.

**Figure:**
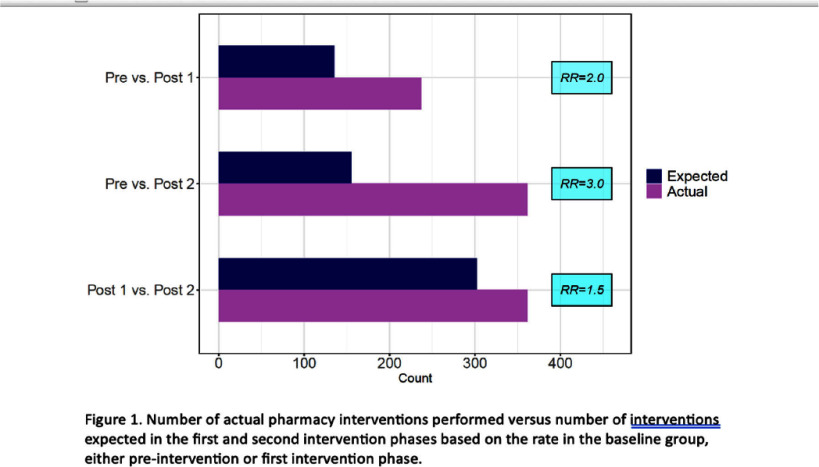

**Figure:**
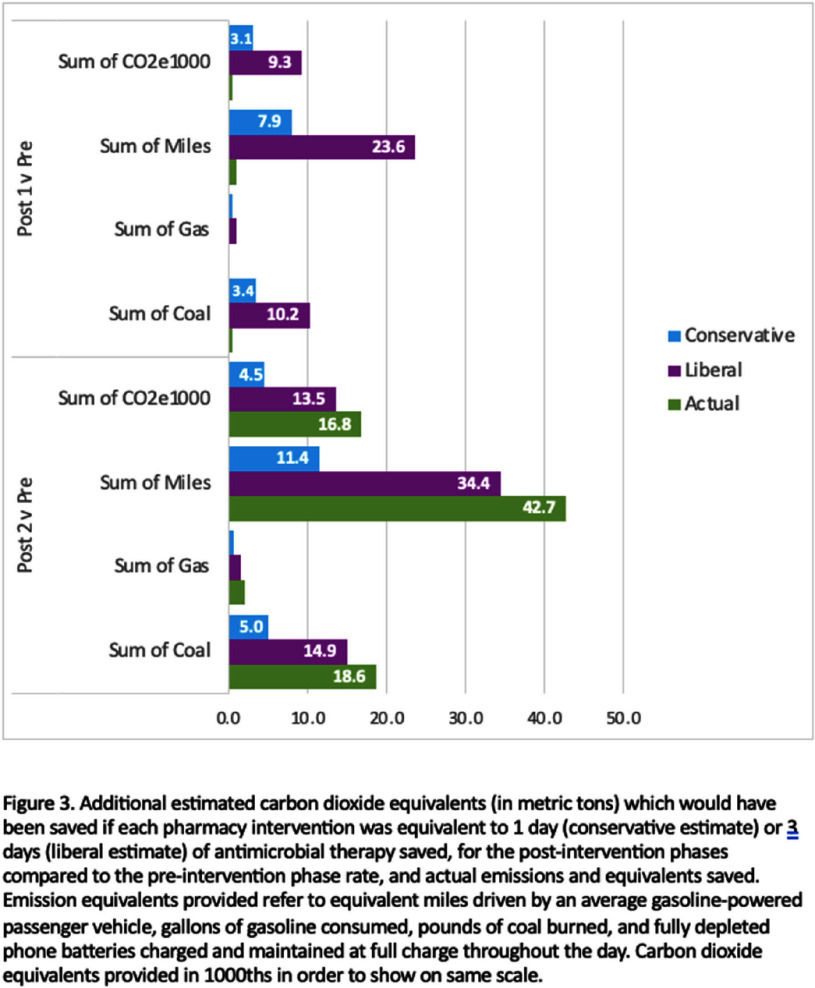

**Figure:**